# Impact of Frailty Risk on Oral Intake and Length of Hospital Stay in Older Patients with Pneumonia: A Historical Cohort Study

**DOI:** 10.3390/jcm12010077

**Published:** 2022-12-22

**Authors:** Shinsuke Hori, Yoshinori Yamamoto, Kenta Ushida, Yuka Shirai, Miho Shimizu, Yuki Kato, Akio Shimizu, Ryo Momosaki

**Affiliations:** 1 Department of Rehabilitation, Mie University Hospital, Tsu 514-8507, Japan; 2 Department of Rehabilitation Medicine, Mie University Graduate School of Medicine, Tsu 514-8507, Japan; 3 Department of Nutrition, Hamamatsu Medicine University Hospital, Hamamatsu 431-3192, Japan; 4 Department of Health Science, Faculty of Health and Human Development, The University of Nagano, Nagano 380-8525, Japan

**Keywords:** hospital frailty risk score, frailty, historical cohort study, pneumonia

## Abstract

The aim of this study was to examine the association between frailty risk and outcomes in older patients with pneumonia. For this purpose, the JMDC multi-center database was used, and a historical cohort study was conducted to examine the association between the Hospital Frailty Risk Score (HFRS) and oral intake prognosis and length of hospital stay in older patients hospitalized with pneumonia. Patients were classified into low-risk (HFRS < 5), intermediate-risk (HFRS = 5–15), and high-risk (HFRS > 15) groups based on their HFRS scores, and outcomes were defined as the number of days from admission to the start of oral intake and length of hospital stay. A total of 98,420 patients with pneumonia (mean age 82.2 ± 7.2) were finally included. Of these patients, 72,207 (73.4%) were in the low-risk group, 23,136 (23.5%) were in the intermediate-risk group, and 3077 (3.1%) were in the high-risk group. The intermediate- and high-risk groups had a higher number of days to the start of oral intake than the low-risk group (intermediate-risk group: coefficient 0.705, 95% confidence interval [CI] 0.642–0.769; high-risk group: coefficient 0.889, 95% CI 0.740–1.038). In addition, the intermediate- and high-risk groups also had longer hospital stays than the low-risk group (intermediate-risk group: coefficient 5.743, 95% CI 5.305–6.180; high-risk group: coefficient 7.738, 95% CI 6.709–8.766). Overall, we found that HFRS is associated with delayed initiation of oral intake and prolonged hospital stay in older patients with pneumonia. Therefore, evaluation based on HFRS could be helpful in making clinical decisions regarding the selection of feeding strategies and when to discharge older patients with pneumonia.

## 1. Introduction

Pneumonia is a common disease that adversely affects the quality of life and prognosis of older patients. In the United States, 5.6 million cases of pneumonia are reported each year, leading to annual health care costs in excess of USD 10 billion [1]. It has been reported that pneumonia and death rates increase with age [2,3], and older patients hospitalized with pneumonia often have difficulty with oral intake [4] and longer hospital stay.

Frailty is a decline in multiple physiological functions associated with aging that is associated with adverse outcomes, including disability, hospitalization, reduced quality of life, and death [5,6,7,8]. Frailty is a well-known complication in older patients with pneumonia [9]. In older patients with pneumonia, frailty linked to re-hospitalization and disability [10]. In addition, frailty is reportedly associated with death and a decline in physical function in older patients hospitalized for pneumonia [11]. It is possible that identifying frailty in older patients with pneumonia may be beneficial in the management of these patients. Frailty is generally assessed using Cardiovascular Health Study criteria based on the phenotype model together with the frailty index based on the accumulated deficit model [6,12]; however, these models are problematic in that they are too complex to use in acute care settings and require a face-to-face evaluation process [13]. The Hospital Frailty Risk Score (HFRS), developed by Gilbert et al., is an evaluation measure of frailty risk using a weighted score based on codes of the International Statistical Classification of Diseases 10th revision (ICD-10) [13]. It has been shown that the HFRS has high concordance with scores on common frailty rating scales and can predict adverse events, such as death and re-hospitalization during hospitalization, in patients with heart failure, pneumonia, hip fracture, vertebral compression fracture, and COVID-19, which are common in older patients [14,15,16,17].

In older patients with aspiration pneumonia, longer duration of difficulty in oral intake is reportedly associated with longer duration of pneumonia treatment and decreased swallowing function [18]. On the other hand, early oral intake in older patients with pneumonia is reported to shorten hospital stay and improve oral intake status [19]. Therefore, management to shorten the duration of oral intake difficulty in older patients with pneumonia is important for the improving their prognosis; however, in clinical practice, it is sometimes difficult to predict early initiation of oral intake for patients with pneumonia. If we can predict the length of time to start of oral intake in older patients with pneumonia, we may be able to provide individualized interventions for early oral intake. We hypothesized that the HFRS on admission could be used to predict early initiation of oral intake in older patients with pneumonia. The aim of this study was therefore to assess the utility of the HFRS in predicting the initiation of oral intake in older patients with pneumonia using a multi-center database.

## 2. Methods

### 2.1. Study Design and Participants

This study comprises a historical cohort study of older patients with pneumonia enrolled in the JMDC multi-center database. As this database is widely used for research and has established academic value, the Ethics Committee of Mie University determined that ethical review was not required. In addition, as this database includes de-identified data that does not contain personal information, informed consent was waived.

We included older patients, defined as aged 65 years or older, who were hospitalized with a diagnosis of pneumonia (ICD-10 codes: S720, S721) between April 2014 and August 2020. Patients with missing Barthel index (BI) or A-DROP data were excluded.

### 2.2. Data Source

The JMDC database includes data on medical reimbursement [20], as well as DPC (Diagnostic Procedure Combination) data. DPC is a medical payment system introduced in Japan in 2003 [21]. The DPC database includes clinical information about the patient, diagnosis, performed surgeries and procedures, medications, and special reimbursement for specific diseases [22]. In addition, the database includes age, gender, A-DROP (as severity of pneumonia) [23], level of consciousness, body mass index (BMI), BI, ventilator use or not, use of vasopressor or not, length of hospital stay, number of days from admission to start of oral intake, number of beds, comorbidity based on ICD-10 codes, and year of admission. The A-DROP is a severity rating scale for pneumonia consisting of age, dehydration, respiratory failure, impaired consciousness, and hypotension [23], where a higher A-DROP score is indicative of more severe pneumonia. Level of consciousness is assessed using the Japan Coma Scale (JCS). The JCS is a widely used consciousness level assessment tool in Japan, consisting of alert (0), dull (1-digit codes: 1, 2, 3), somnolence (2-digit codes: 10, 20, 30), and coma (3-digit codes: 100, 200, 300) [24]. BMI is calculated by dividing weight (kg) by the square of height (m) and is used to assess obesity or underweight status. BMI was categorized as <18.5, 18.5–24.9, 25.0–29.9, and 30.0 or higher [25]. BI was used to assess the activities of daily living (ADL) in patients. Higher BI scores indicate greater independence in ADL [26]. The age of the patient was classified as pre-old age (65–74 years), old age (75–89 years), and oldest-old (90 years or older) based on the definitions of the Japanese Geriatrics Society [27]. We addressed the use of ventilators and vasopressor on admission.

### 2.3. HFRS

The HFRS assesses patient frailty based on ICD-10 codes. The HFRS was calculated retrospectively based on all ICD-10 codes available at the time of admission. Each of these ICD-10 diagnostic codes was assigned a specific value proportional to its strength in predicting frailty, e.g., the highest score of 7.1 was for ICD code F00 (Alzheimer’s disease). Details of the HFRS calculation can be found in the original HFRS study by Gilbert et al. [13]. Patients were categorized into low-risk (HFRS < 5), medium-risk (HFRS 5–15), and high-risk (HFRS > 15) groups according to previous studies [16].

### 2.4. Outcome

The primary outcomes were the number of days from hospitalization to start of oral intake and length of hospital stay. The start of oral intake was determined by the provision of food from the hospital. Additional secondary outcomes were difficulty starting oral intake within 3 and 5 days from admission and hospital stay of 15 days or more and of 30 days or more.

### 2.5. Statistical Analysis

We compared baseline information and outcomes between low-, intermediate-, and high-risk groups. Categorical data are presented as absolute values and percentages, and χ^2^ tests were used to compare proportions among the three groups. Continuous data are expressed as mean ± standard deviation, and differences among the three groups in frailty risk were analyzed by one-way analysis of variance. Multiple regression analysis was conducted to analyze the association between HFRS and the number of days from hospitalization to start of oral intake and hospital stay. Multiple logistic regression analysis was conducted to examine the associations between HFRS, delayed start of oral intake, and prolonged hospitalization. The covariates in the multi-variate analysis were age, sex, A-DROP, JCS at admission, BMI, BI at admission, ventilator use or not, vasopressor use or not, number of beds, and year of admission. Statistical analysis was performed using SPSS software (version 26.0, IBM Japan, Tokyo, Japan). The statistical significance level was set at *p* < 0.05.

## 3. Results

**Figure 1:**
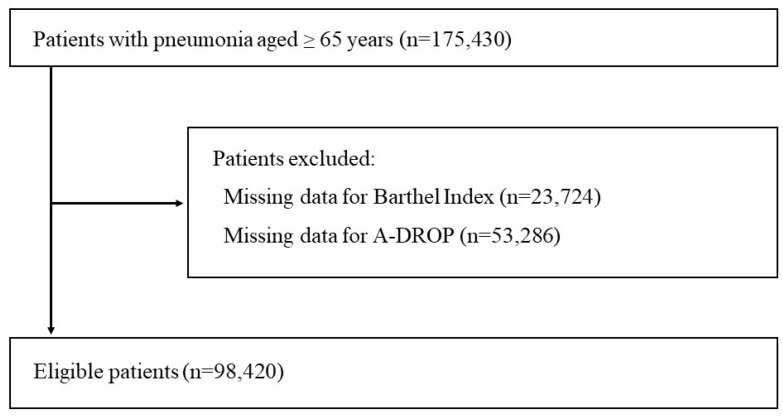
Overview of patient selection.

Within the 175,430 patients aged 65 years or older hospitalized for pneumonia, 77,010 were excluded due to missing BI scores (23,724 patients) or missing A-DROP data (53,286 patients). In total, we included 98,420 patients with pneumonia in the analysis (Figure 1).

Patients were categorized into low-risk (72,207; 73.4%), intermediate-risk (23,136; 23.5%), and high-risk (3077; 3.1%) groups based on their HRFS. Table 1 shows the patient background data. Patients categorized into the high-risk group were more likely to be female (55.9%), older than 90 years (38.3%), underweight (38.3%), and had a lower BI on admission (13.3 ± 26.4) than patients in the other frailty risk groups.

Table 2 shows the results for comparison of outcomes between the groups. Frailty risk is found to be significantly associated with the number of days from admission to start of oral intake, length of hospital stay, delayed start of oral intake, and long-term hospitalization.

Table 3 shows the results of the multiple regression analysis on outcomes. After adjustment for confounding factors, a positive correlation was observed between frailty risk and days to start of oral intake (intermediate-risk group: coefficient 0.705, 95% confidence interval [CI], 0.642–0.769; high-risk group: coefficient 0.889, 95% CI, 0.740–1.038). In addition, a positive correlation with length of hospital stay (intermediate-risk group: coefficient 5.743, 95% CI, 5.305–6.180; high-risk group: coefficient 7.738, 95% CI, 6.709–8.766) was observed.

Table 4 shows the results of the multiple logistic regression analysis. The higher the frailty risk, the less likely the patient was to start oral intake within 3 days of admission (intermediate-risk group: odds ratio [OR] 1.631, 95% CI 1.560–1.704; high-risk group: OR 1.756, 95% CI 1.606–1.921). The higher the frailty risk, the less likely they were to start oral intake within 5 days of admission (intermediate-risk group: OR 1.644, 95% CI 1.559–1.733; high-risk group: OR 1.733, 95% CI 1.561–1.923). Furthermore, the higher the frailty risk, the higher the OR of long-term hospitalization for more than 15 days (intermediate-risk group: OR 1.615, 95% CI 1.563–1.668; high-risk group: OR 1.979, 95% CI 1.826–2.145) and more than 30 days (intermediate-risk group: OR 1.613, 95% CI 1.556–1.672, *p* < 0.001; high-risk group: OR 1.628, 95% CI 1.504–1.762, *p* < 0.001).

## 4. Discussion

We used a multi-center database to examine the association between frailty risk based on HFRS and prognosis in older patients with pneumonia. We found that frailty risk is associated with both the number of days from admission to start oral intake and the length of hospital stay. Patients in the intermediate- or high-risk groups were less likely to start of oral intake within 3 and 5 days and had higher rates of prolonged hospitalization (i.e., of more than 15 or 30 days) compared with patients in the low-risk group.

In previous studies, HFRS was reported to be related to functional outcomes in various diseases. Kundi et al. [14] reported associations between HFRS, death, and re-hospitalization in heart failure patients. Shimizu et al. [15,16] also reported an association between HFRS and in-hospital mortality in patients with hip fracture and vertebral compression fracture. There are reports of HFRS being related to death and re-hospitalization in patients hospitalized for pneumonia [14], although there are no studies reporting its association with the number of days to start of oral intake or length of hospital stay from admission. To the best of our knowledge, this is the first study to examine the association between HFRS and the number of days to the start of oral intake and hospital stay in older patients with pneumonia.

There are several possible explanations for the higher number of days to the start of oral intake and longer hospital stay in older patients with pneumonia who have intermediate- or high-risk HFRS. It has been reported that frailty is associated with poor swallowing function in older patients [4] and that patients with an HFRS indicating intermediate or higher risk may originally have had poor swallowing function, delaying the start of oral intake. A delay in the start of oral intake may also delay the establishment of feeding methods, leading to a delay in clinical decisions regarding clinical prognosis and patient discharge. Therefore, patients with higher HFRS and a delayed start of oral intake may have longer hospital stays.

The present study involved many older adults who originally had a low BMI, and it is likely that many of them had low swallowing function. Patients with pneumonia who do not originally have much decline in swallowing function are likely to be able to start oral intake immediately after admission, and the results of this study may not be applicable to them. In addition, there were relatively mild cases of pneumonia in this study. In severe pneumonia, the damage caused by pneumonia is expected to be greater than the original risk of frailty, and the results of this study may not be applicable.

Older patients with pneumonia are more likely to have difficulty starting oral intake after hospitalization, and it is often difficult to deal with this problem. Maeda et al. reported that more than 60% of pneumonia patients were not taking food orally on the third day after admission, and 35% were still not starting oral intake 7 days after admission [28]. Patients with aspiration pneumonia who do not have oral intake reportedly have prolonged treatment duration and decreased swallowing function [18]. Early decision making on how to feed the patient after hospitalization for pneumonia seems to be important, and early prediction of difficulty in starting oral intake may lead to early and appropriate clinical decisions regarding the choice of feeding method and when to discharge the patient from the hospital.

High-risk HFRS groups are expected to have difficulty with oral intake, and it may be useful to take appropriate action depending on the score. First, older patients with pneumonia may be more likely to have poor oral hygiene after a long period of oral intake inability [29]. It may be beneficial to engage dental-related professionals to maintain a good oral environment. Additionally, prolonged periods of oral intake inability may worsen the nutritional status; therefore, it may be advisable to have a dietitian manage the nutritional status. If the patient has difficulty achieving oral intake independence, it may be difficult to discharge the patient home; therefore, it may be beneficial to have a social worker involved at an early stage to help coordinate discharge from the hospital.

This study has several limitations. First, the data used in the study were limited to those available from the hospital information system. Therefore, it was not possible to obtain information on swallowing function, cognitive function, pre-hospitalization oral intake status, and living situation, which might be associated with the start of oral intake in older patients with pneumonia. Second, as the HFRS is based on ICD-10 codes, the coding accuracy could pose an issue. However, the validity of ICD-10 codes in the DPC database for diagnosis of electronic medical records has been verified [22], and we believe that any problem due to coding accuracy would not be a major issue. Third, in February 2022, a new version of ICD-11 was published [30]; however, our study did not use ICD-11 codes. The DPC database in Japan includes only ICD-10 codes, and it was not possible to calculate the HFRS using ICD-11 codes. In the future, when ICD-11 becomes more widely used, it might be required to calculate the HFRS using ICD-11 codes. Fourth, this study had a long observation period of 6 years, and it is possible that the treatment may have changed over time. However, to the best of our knowledge, there have been no new treatment techniques introduced, or reimbursement schemes or clinical guidelines published in the past 6 years, which would have a significant impact on the treatment of older patients with pneumonia. Even if there had been changes that would have affected patients, we believe that the impact would have been taken into account because we adjusted for the year of hospitalization.

## 5. Conclusions

In this study, we found that HFRS calculated using medical information data is related to delayed start of oral intake and longer length of hospital stay in older patients with pneumonia. The obtained results may be useful in informing clinical decisions regarding the selection of feeding methods and time of discharge for older patients with pneumonia; however, further research is still needed to validate the use of HFRS.

## Figures and Tables

**Table 1:** Patient characteristics.

	Low-Risk Group (Hospital Frailty Risk Score < 5)	Intermediate-Risk Group (Hospital Frailty Risk Score = 5–15)	High-Risk Group (Hospital Frailty Risk Score > 15)	*p*-Value
Number of patients, *n* [%]	72,207 [73.4]	23,136 [23.5]	3077 [3.1]	
Female sex, *n* [%]	30,621 [42.4]	11,375 [49.2]	1720 [55.9]	<0.001
Age, years, *n* [%]				<0.001
- 65–74	15,431 [21.4]	2203 [9.5]	150 [4.9]	
- 75–89	39,452 [54.6]	12,994 [56.2]	1748 [56.8]	
- ≥90	17,324 [24.0]	7939 [34.3]	1179 [38.3]	
A-DROP, *n* [%]				<0.001
- 0	16,934 [23.5]	2753 [11.9]	344 [11.2]	
- 1	28,076 [38.9]	7588 [32.8]	945 [30.7]	
- 2	17,825 [24.7]	7277 [31.5]	971 [31.6]	
- 3	6851 [9.5]	3895 [16.8]	558 [18.1]	
- 4	2073 [2.9]	1397 [6.0]	228 [7.4]	
- 5	448 [0.6]	226 [1.0]	31 [1.0]	
Body mass index, *n* [%]				<0.001
- <18.5	22,545 [31.2]	8625 [37.3]	1178 [38.3]	
- 18.5–25	31,510 [43.6]	8428 [36.4]	973 [31.6]	
- 25.0–30.0	7472 [10.3]	1539 [6.7]	181 [5.9]	
- ≥30.0	1276 [1.8]	257 [1.1]	30 [1.0]	
- Missing	9404 [13.0]	4287 [18.5]	715 [23.2]	
Barthel index at admission, mean ± SD	52.4 ± 41.7	22.8 ± 33.7	13.3 ± 26.4	<0.001
Ventilator use on admission, *n* [%]	1464 [2.0]	516 [2.2]	31 [1.0]	<0.001
Vasopressor use on admission, *n* [%]	1389 [1.9]	619 [2.7]	68 [2.2]	0.003
Number of beds, *n* [%]				<0.001
- 20–99	3459 [4.8]	1121 [4.8]	317 [10.3]	
- 100–199	23,217 [32.2]	6726 [29.1]	895 [29.1]	
- 200–299	13,508 [18.7]	4725 [20.4]	642 [20.9]	
- 300–499	22,684 [31.4]	7942 [34.3]	909 [29.5]	
- ≥500	9339 [12.9]	2622 [11.3]	314 [10.2]	
Year of admission, *n* [%]				<0.001
- 2014	4863 [6.7]	1219 [5.3]	106 [3.4]	
- 2015	8122 [11.2]	1994 [8.6]	203 [6.6]	
- 2016	10,514 [14.6]	3271 [14.1]	418 [13.6]	
- 2017	12,478 [17.3]	4386 [19.0]	624 [20.3]	
- 2018	14,472 [20.0]	4821 [20.8]	621 [20.2]	
- 2019	15,901 [22.0]	5246 [22.7]	760 [24.7]	
- 2020	5857 [8.1]	2199 [9.5]	345 [11.2]	

**Table 2:** Comparison of Hospital Frailty Risk Score and outcomes between groups.

	Low-Risk Group (Hospital Frailty Risk Score < 5)	Intermediate-Risk Group (Hospital Frailty Risk Score = 5–15)	High-Risk Group (Hospital Frailty Risk Score > 15)	*p*-Value
Number of days from hospitalization to start of oral intake, mean ± SD	1.2 ± 3.3	2.8 ± 5.5	3.2 ± 5.5	<0.001
Difficulty starting oral intake within 3 days from admission, *n* [%]	6395 [9.4]	5048 [24.0]	827 [29.3]	<0.001
Difficulty starting oral intake within 5 days from admission, *n* [%]	3756 [5.5]	3233 [15.3]	535 [19.0]	<0.001
Length of hospital stay, mean ± SD	19.7 ± 25.5	29.1 ± 35.0	32.3 ± 40.9	<0.001
Hospital stay ≥15 days, *n* [%]	30,457 [42.2]	14,436 [62.4]	2144 [70.0]	<0.001
Hospital stay ≥30 days, *n* [%]	12,037 [16.7]	7303 [31.6]	1061 [34.5]	<0.001

**Table 3:** Multiple linear regression analysis for primary outcomes.

Variables	Coefficient	95% Confidence Interval		*p*-Value
		Lower	Upper	
Number of days from hospitalization to start of oral intake				
Low-risk group (reference)	―	―	―	
Intermediate-risk group	0.705	0.642	0.769	<0.001
High-risk group	0.889	0.740	1.038	<0.001
Length of hospital stay				
Low-risk group (reference)	―	―	―	
Intermediate-risk group	5.743	5.305	6.180	<0.001
High-risk group	7.738	6.709	8.766	<0.001

Models adjusted for gender, age, A-DROP, body mass index at admission, Japan Coma Scale at admission, Barthel index on admission, ventilator use on admission, vasodilator use on admission, number of beds, and year of admission.

**Table 4:** Multiple logistic regression analysis for secondary outcomes.

Variables	Odds Ratio	95% Confidence Interval		*p*-Value
		Lower	Upper	
Difficulty starting oral intake within 3 days from admission				
Low-risk group (reference)	―	―	―	
Intermediate-risk group	1.631	1.560	1.704	<0.001
High risk-group	1.756	1.606	1.921	<0.001
Difficulty starting oral intake within 5 days from admission				
Low-risk group (reference)	―	―	―	
Intermediate-risk group	1.644	1.559	1.733	<0.001
High-risk group	1.733	1.561	1.923	<0.001
Hospital stay ≥15 days				
Low-risk group (reference)	―	―	―	
Intermediate-risk group	1.615	1.563	1.668	<0.001
High-risk group	1.979	1.826	2.145	<0.001
Hospital stay ≥30 days				
Low-risk group (reference)	―	―	―	
Intermediate-risk group	1.613	1.556	1.672	<0.001
High-risk group	1.628	1.504	1.762	<0.001

Models adjusted for gender, age, A-DROP, body mass index at admission, Japan Coma Scale at admission, Barthel index on admission, ventilator use on admission, vasodilator use on admission, number of beds, and year of admission.

## Data Availability

Data sharing not applicable—no new data generated.
